# Esophageal impedance baseline according to different time intervals

**DOI:** 10.1186/2047-783X-17-18

**Published:** 2012-06-19

**Authors:** Dario Ummarino, Silvia Salvatore, Bruno Hauser, Annamaria Staiano, Yvan Vandenplas

**Affiliations:** 1 Department of Pediatrics, Universitair KinderZiekenhuis Brussel, Vrije Unversiteit Brussel, Laarbeeklaan, 101, Brussels, 1090, Belgium; 2 University of Naples Federico II, Naples, Italy; 3 Clinica Pediatrica, Università dell’Insubria, Varese, Italy

**Keywords:** Esophageal impedance baseline, Multichannel intraluminal impedance, pH monitoring, Time intervals, 24 h tracing

## Abstract

**Background:**

The impedance baseline has been shown to reflect esophageal integrity, and to be decreased in patients with esophagitis. However, different methods for the determination of the impedance baseline have not been compared.

**Methods:**

The median impedance baseline was calculated in 10 consecutive multichannel intraluminal impedance recordings in children with non-erosive reflux disease. All children underwent an endoscopy with a biopsy as part of the clinical work-up to exclude esophagitis. The impedance baseline was obtained both by including and excluding all impedance episodes (IE; reflux, swallows and gas episodes) during the full recording, and during the first 1-minute period without an IE every hour (method 1), every 2 hours (method 2) or every 4 hours (method 3). The impedance baseline obtained during the full recording was set at 100%, and the variation (difference in impedance baseline for the different methods) and variability (difference in impedance baseline during one analysis period) were assessed.

**Results:**

None of the participants had esophagitis. The mean difference over the six channels between the impedance baseline over the total recording with and without IE was approximately 2.5%, and comparable for each channel (range 0.47% to 5.55%). A mean of 1,028 IEs were excluded in each tracing, and it took between 4 and 24 hours to delete all events in one tracing. The difference in the impedance baseline obtained with and without IEs was mainly caused by the gas episodes in the upper channels and swallows in the lower channels. The median impedance baseline according to the three one-minute analysis methods was comparable to the median impedance baseline according to the 24 hour analysis.

**Conclusions:**

The automatic determination of the median impedance baseline over the total tracing including IEs is an adequate method. In isolated tracings with numerous IEs, the calculation of the median impedance baseline over one minute every 4 hours is an alternative option. Companies should develop software to calculate the median impedance baseline during the whole registration deleting all IEs for the analysis.

## Background

Multichannel intraluminal impedance (MII), the inverse measurement of intraluminal conductivity, detects the flow of luminal contents [1]. When impedance is measured along a segment of the esophageal lumen using an array of impedance electrodes, the passage of a bolus of high conductivity (liquid content) produces a propagated decrease in the impedance from baseline. Conversely, the passage of a bolus of low conductivity (air) produces a propagated increase in the impedance from baseline [2]. An additional pH-sensitive electrode allows the differentiation between acid and non-acid reflux. According to the North American Society for Pediatric Gastroenterology, Hepatology, and Nutrition/European Society for Pediatric Gastroenterology, Hepatology, and Nutrition guidelines, MII-pH recording is superior to pH monitoring alone to diagnose gastroesophageal reflux disease (GERD) [3]. In the period of time during which no substance passes through the esophageal lumen, the esophagus is collapsed and the level of impedance measured represents the inverse conductivity of the esophageal mucosa [4].

The impedance baseline (IB) has recently been reported to reflect esophageal mucosal integrity. Farre *et al*. evaluated the IB *in vivo* and *in vitro* in rabbits and healthy human participants, and reported that mucosal damage after esophageal acid exposure causes a drop in the IB. In rabbits, the IB is decreased when the esophagus is exposed to acid by about one-third compared with healthy controls [5]. Measurement of the IB may increase the information obtained from a MII-pH recording. However, the method of calculating the IB has not been standardized in the literature. Therefore, we compared different methods to obtain a reliable and easy to calculate the IB.

## Methods

We analyzed 10 consecutive MII-pH tracings performed in children with symptoms suggesting GERD (regurgitation or vomiting, food refusal, irritability, crying, failure to thrive, abdominal pain, heartburn) but without endoscopic or histologic esophagitis. All patients underwent an endoscopy with a biopsy as part of the clinical work-up to exclude esophagitis. The MII-pH was performed as part of the clinical work-up; parents and/or children gave their written consent to use the data of the recording for scientific purposes Approval of the local ethical committee was obtained. The MII-pH recording was performed with a portable data logger (50 Hz) and a combined impedance-pH catheter (Ohmega Ambulatory Impedance-pH Recorder, MMS impedance device, MMS, Inc.). The probe was placed transnasally; the location of the probe was determined with fluoroscopy with the pH sensor at the second vertebra above the diaphragm. An infant or pediatric probe was used according to the patient’s height (below or above 75 cm, respectively). Impedance rings were 1.5 cm or 2 cm apart from each other in the infant and pediatric catheter, respectively. The recordings were performed when the participants were ambulatory, and they were encouraged to maintain normal activities, including sleep time and meals.

Impedance data were analyzed using a dedicated software program (MMS Analysis software, MMS, Inc.) and visual analysis. In addition, the IB was determined in every tracing through a specific option of the MMS software. Gas reflux was defined as a rapid increase in impedance >3,000 ohms, occurring simultaneously in at least two segments. A liquid reflux episode was defined when a fall in impedance of more than or equal to 50% from baseline occurred in at least two consecutive channels in an aboral direction. Swallows were defined as a drop in impedance starting at the highest channel and going in the antegrade direction to a reflux episode [6].

We calculated the median esophageal IB with different methods. First, the IB was determined over the whole recording, including all impedance events (IE; gas, liquid, mixed reflux and swallows). A second determination was done over the entire recording period, but now excluding all IEs. The time spent for this analysis was recorded. Subsequently, three different ‘one-minute’ methods were applied. The median IB was determined during the first ‘stable minute’ (one minute without a reflux episode or swallow) every hour (first method), every 2 hours (second method) or every 4 hours (third method).

We postulated that the IB obtained during the total (20 to 24 h) recording including all IEs equaled 100%, and we compared the values obtained over 24 h with and without IE and the results of the three one-minute methods, obtained by the mean of the different one-minute values. The difference was expressed as a percentage. We calculated the variation (the difference in the IB according to the different methods) and variability (the difference in the IB obtained with one method). The smaller the variation and variability, the better. Positive and negative values reflect higher and lower results compared to the total recording. A *t*-test was used to test statistical significance; a *P-*value <0.05 was considered significant.

## Results

No patient had endoscopic or histologic esophagitis. The mean age ± SD of the study population was 63 ± 36 months (range 17 to 121 months). A mean of 1,028 IEs (135 gas, 81 reflux (acid and non-acid) and 812 swallows) were found per tracing and were excluded in the analysis without IEs. The manual exclusion of these events took an average of 8 hours (range 4 to 24 hours). The median IB and the range according to the two methods are reported in Tables 1 and 2. Overall, the difference in the IB including or excluding all IEs was evaluated in every patient and the results was statistically not significant (*P =* 0.55) (Table 2). The difference in the IB by comparing with and without IEs was mainly caused by gas episodes in the upper channels and swallows in the lower channels.

**Table 1:** Basal impedance: median, minimum and maximum including or excluding all impedance events

	**24 h without exclusion of IE**			**24 h with exclusion of IE**		
	**Median**	**Minimum**	**Maximum**	**Median**	**Minimum**	**Maximum**
Channel 1	4232,3	2652,0	7753,0	3933,4	2642,0	4846,0
Channel 2	3803,2	2572,0	4737,0	3836,8	2526,0	5102,0
Channel 3	3464,7	2391,0	4499,0	3533,5	2347,0	4438,0
Channel 4	3514,0	2484,0	4372,0	3610,4	2684,0	4565,0
Channel 5	3754,9	2719,0	5136,0	3952,6	2776,0	4953,0
Channel 6	3536,0	2183,0	4799,0	3715,9	2223,0	5013,0

*Legend:* IE: impedance event.

**Table 2:** Difference in median basal impedance (%) including and excluding all impedance events over the whole recording

	**Median**	**Minimum**	**Maximum**
Channel 1	- 2.92	- 48.60	5.71
Channel 2	0.47	- 8.68	9.44
Channel 3	2.07	- 8.82	11.99
Channel 4	3.27	- 7.35	14.81
Channel 5	5.55	- 7.52	17.56
Channel 6	5.14	−7.56	17.65

The data refer to the median of the values obtained in every patient.

## Discussion

Recent studies reported a decreased the IB in (adult) patients with esophageal acid exposure and esophagitis compared with healthy volunteers [6-8]. These findings suggest that the IB could be a marker of reflux-induced changes of the esophageal mucosa [6]. The IB may thus provide interesting information about mucosal integrity. The aim of our study was to evaluate the best method to determine the IB. The easiest way is to use the (automatic) software function which analyses the mean of all impedance values in each channel during the total recording time. This method has been applied before [9]. However, this method integrates all the swallows, gas and bolus reflux episodes. In theory, the IB seems be best calculated over the total registration period excluding all IE. Since swallows and reflux episodes are characterized by a decrease in impedance, and gas refluxes are defined as a rise in impedance, the more IEs occur, the more that baseline will be influenced. Since elimination of all IEs over a 24 hour registration is time consuming, we tested if an IB determined over shorter time intervals would provide comparable results. Most authors calculate only one IB (based on all channels, or on the most distal channels). We could demonstrate that the IB is higher in the more proximal channels (4,232 Ohm) compared with the distal channel (3,536 Ohm).

Several studies reported previously on the IB, but used different methods. In adults affected by achalasia, Nguyen *et al*. evaluated the IB analyzing a period of time as short as 4 seconds [10]. In preterm infants, López-Alonso *et al*. reported that the IB value in the distal esophagus was 1,750 (interquartile range 1,500 to 2,050), but the method used to calculate IB was not specified [9]. Loots *et al*. evaluated the IB in children before and after proton-pump inhibitor therapy after deleting confounding effects caused by gas (but not liquid) reflux by excluding all data > 5,000 Ω [11].

We compared first the median IB obtained by the ‘raw’ tracings with the IB obtained after exclusion of all IEs (liquid and gas reflux episodes, and swallows). IB with or without IEs differs by −3% in channel 1 and 5% in channel 6. The elimination of all IEs would thus result in a more accurate median IB, but is more time consuming, as it took a minimum of 4 hours and a maximum of 24 hours to delete all IEs in a single tracing. Nowadays, it is technically impossible to exclude IEs with an automatic tracing analysis. In other words, the best theoretical method (calculate the IB of the whole recording deleting all IEs) is not applicable in practice. Therefore, we hypothesized that calculation of the IB over shorter periods may provide reliable results. We calculated the median IB during one-minute intervals without any IEs and compared this value with the 24-hour results with and without IEs. The median IB according to the three one-minute methods results was comparable and the difference of the mean in the six channels was less than 3% (Table 2). Compared to the median IB over the 24 hours, the maximal difference was 5.44% (Table 3). Conversely, variability during a one-minute period was high. The variability in channel 1 seems mainly caused by air. The small number of patients, which is due to the time needed to eliminate all relevant impedance events from the tracing, is a weakness of this analysis.

**Table 3:** Median, minimum and maximum values obtained according to the three one-minute methods

	**Method 1**			**Method 2**			**Method 3**		
	**Median**	**Minimum**	**Maximum**	**Median**	**Minimum**	**Maximum**	**Median**	**Minimum**	**Maximum**
Channel 1	3,916.4	2,664.4	4,861.1	3,836.1	2,719.3	4,533.9	3,906.3	2,848.0	5,098.2
Channel 2	3,788.9	2,504.7	4,766.6	3,721.6	2,611.1	4,812.3	3,805.3	2,655.2	4,847.2
Channel 3	3,443.1	2,328.2	4,674.6	3,468.5	2,363.0	4,756.4	3,565.5	2,513.0	5,463.0
Channel 4	3,501.5	2,434.6	4,532.4	3,508.0	2,291.0	4,945.5	3,470.1	2,124.7	5,188.0
Channel 5	3,859.0	2,757.0	5,535.2	3,833.5	2,642.8	5,862.3	3,770.6	2,287.7	5,619.2
Channel 6	3,567.7	2,065.3	4,886.5	3,517.7	1,967.5	4,892.4	3,510.4	1,992.7	4,910.8

Median basal impedance according to the first stable minute without impedance event, every hour (method 1), every 2 hours (method 2) and every 4 hours (method 3).

The median and range of the three one-minute methods are shown in Table 3. The variation in the IB according to the three methods was small (mean 2.5%, range 0.47% to 5.55%) (Table 4), but the variability was high (up to 45.5%) (Table 5). The difference in the median IB between the one-minute methods seems not to differ if compared with the IB over the total registration when excluding or maintaining all IEs (Tables 2 and 6). The high variability indicates that the IB is not really stable during a period of one minute without IE throughout the registration time. Variability is largest with method 3.

**Table 4:** Variation of the three one-minute methods according to median impedance baseline over the 24 hour recording, with and without impedance events

		**Method 1 (%)**	**Method 2 (%)**	**Method 3 (%)**
Channel 1	24 h with IE	−3.77	−5.38	−3.89
	24 h without IE	−0.35	−2.06	−0.14
Channel 2	24 h with IE	−0.69	−2.49	0.24
	24 h without IE	−0.93	−2.69	0.14
Channel 3	24 h with IE	−0.71	−0.11	2.79
	24 h without IE	−2.39	−1.78	1.26
Channel 4	24 h with IE	−0.28	−0.67	−2.18
	24 h without IE	−2.94	−3.35	−4.97
Channel 5	24 h with IE	2.59	1.53	0.16
	24 h without IE	−2.30	−3.15	−4.21
Channel 6	24 h with IE	0.44	−0.98	−0.87
	24 h without IE	−4.06	−5.44	−5.15

Variation expresses the difference in the impedance baseline according to the different methods. Negative values mean that the values obtained with the method are lower than that obtained during the 24 h recording. IE: impedance event.

**Table 5:** Variability according to the three one-minute methods compared with the values obtained during to the 24 h analysis with and without impedance events

		**Method 1 (%)**		**Method 2 (%)**		**Method 3 (%)**	
		**Maximum**	**Minimum**	**Maximum**	**Minimum**	**Maximum**	**Minimum**
Channel 1	24 h with IE	5.47	−44.10	5.35	−45.51	9.19	−40.35
	24 h without IE	8.76	−9.43	6.01	−10.88	16.05	−14.65
Channel 2	24 h with IE	5.34	−5.50	5.77	−9.35	15.60	−9.50
	24 h without IE	9.74	−8.15	10.18	−12.05	20.43	−16.65
Channel 3	24 h with IE	8.86	−11.42	11.62	−15.49	21.43	−9.13
	24 h without IE	6.58	−16.58	14.52	−13.11	24.56	−17.58
Channel 4	24 h with IE	11.07	−11.36	15.17	−17.72	20.85	−38.01
	24 h without IE	11.53	−17.12	21.69	−15.09	26.32	−33.09
Channel 5	24 h with IE	11.87	−12.79	14.14	−10.24	18.52	−24.13
	24 h without IE	11.75	−17.09	18.36	−17.61	21.92	−28.38
Channel 6	24 h with IE	7.14	−6.13	6.50	−9.87	13.53	−24.49
	24 h without IE	11.90	−14.44	8.63	−21.17	22.82	−28.01

Variability expresses the difference in the impedance baseline obtained with one method. Negative values mean that the value obtained with the method is lower than that obtained in the 24 h recording. IE: impedance events.

**Table 6:** Basal impedance: median, minimum and maximum including or excluding all impedance events

	**24 h without exclusion of IE**			**24 h with exclusion of IE**		
	**Median**	**Minimum**	**Maximum**	**Median**	**Minimum**	**Maximum**
Channel 1	4232.3	2652.0	7753.0	3933.4	2642.0	4846.0
Channel 2	3803.2	2572.0	4737.0	3836.8	2526.0	5102.0
Channel 3	3464.7	2391.0	4499.0	3533.5	2347.0	4438.0
Channel 4	3514.0	2484.0	4372.0	3610.4	2684.0	4565.0
Channel 5	3754.9	2719.0	5136.0	3952.6	2776.0	4953.0
Channel 6	3536.0	2183.0	4799.0	3715.9	2223.0	5013.0

IE: impedance event.

## Conclusion

Our results suggest that calculating the IB over a one-minute interval every hour, or every two or four hours, results in an IB that is comparable with the 24-hour median IB. The theoretically most accurate method to calculate the IB would be the result of the IB obtained over the entire recording after elimination of all IEs. However, this method is too laborious to be used in practice. Since the median IB resulting from a 24-hour automatic analysis including all IEs is statistically not different from the theoretically best method, the automatic analysis of the whole tracing is the most logic choice. In isolated tracings with a very high number of IEs, the determination of an IB during a one-minute stable period without IEs every four hours is an acceptable alternative. Companies should develop software to calculate the median IB during the whole registration deleting all IEs for the analysis.

## Competing interests

YVDP is consultant for Biocodex and United Pharmaceuticals. None of the other authors reported any competing interest. The authors declare that they have no competing interest with equipment or software regarding MII-pH monitoring.

## Authors’ contribution

DU collected the data, and took part on writing the manuscript. AS, SS and BH included patients and contributed to the writing of the paper. YVDP co-wrote the manuscript and discussed the results with DU. All authors read and approved the manuscript.
